# From Knowledge to Practice: The Effect of Multimodal Strategies on Hand Hygiene Improvement in Tunisia

**DOI:** 10.3390/tropicalmed10060162

**Published:** 2025-06-11

**Authors:** Maissa Ben Jmaa, Mariem Ben Hmida, Houda Ben Ayed, Hanen Maamri, Maroua Trigui, Nimer Ortuño-Gutiérrez, Aelita Sargsyan, Mondher Kassis, Rony Zachariah, Sourour Yaich

**Affiliations:** 1 Community Health and Epidemiology Department, Hedi Chaker University Hospital, University of Sfax, Sfax 3023, Tunisiahanen.maamri@medecinesfax.org (H.M.); yaich.sourour@gmail.com (S.Y.); 2 Hygiene Department, Hedi Chaker University Hospital, University of Sfax, Sfax 3023, Tunisia; houda.benayed@medecinesfax.org; 3 Hygiene Department, Habib Bourguiba University Hospital, University of Sfax, Sfax 3023, Tunisia; trigui_maroua@medecinesfax.org (M.T.); kassis_mondher@medecinesfax.org (M.K.); 4 Damien Foundation, 1090 Brussels, Belgium; ortunonimer@gmail.com; 5 Tuberculosis Research and Prevention Centre, Yerevan 0033, Armenia; sargsyan.aelita@gmail.com; 6 Research for Implementation, UNICEF/UNDP/World Bank/WHO Special Programme for Research and Training in Tropical Diseases (TDR), World Health Organization, 1211 Geneva, Switzerland; zachariahr@who.int

**Keywords:** quality of care, operational research, SORT IT, SDGs

## Abstract

Hand hygiene reduces healthcare-associated infections. The World Health Organization (WHO) recommends the multimodal hand hygiene strategy to improve hand hygiene. To compare hand hygiene knowledge and compliance of healthcare practitioners (HCPs) before and after the implementation of the WHO multimodal strategy, a before-and-after cross-sectional study was conducted in two Tunisian University Hospitals (2019–2023). Hand hygiene knowledge and compliance were assessed using the WHO questionnaire and observation tool. In 2019, 42 of 246 HCPs (17%) correctly answered ≥80% of 25 questions on hand hygiene knowledge. By 2023, this increased to 47 HCPs (19%). Knowledge on hand hygiene significantly improved for 10 out of 25 questions (12–38% increases) but declined for eight questions (5–40% decreases). Seven questions showed no significant changes in knowledge. Overall hand hygiene compliance increased from 21% in 2019 to 40% in 2023 (*p* < 0.001). Improvements were observed across the hospital departments (*p* < 0.001) and staff categories (*p* < 0.001). In 2023, the lowest hand hygiene compliance was for/before touching a patient (29%), and before clean/aseptic procedures (37%). Hand hygiene compliance was improved, but progress fell short of the WHO’s desired 80% target. Sustained efforts and complementary interventions are needed to accelerate progress and achieve the desired outcomes.

## 1. Introduction

Healthcare-associated infections (HAIs) are a significant global public health problem, contributing to longer hospital stays, long-term disability, considerable morbidity and mortality, an elevated risk of antimicrobial resistance, and higher economic loss [1,2]. The hands of healthcare workers play a critical role in the transmission of microorganisms responsible for HAIs.

Over the last two decades, there has been cumulative evidence demonstrating that hand hygiene is effective in reducing and preventing HAIs [3,4]. In line with global efforts to mitigate the burden of HAIs, significant attention has been directed towards improving hand hygiene, including adoption of the World Health Organization (WHO) multimodal hand hygiene strategy [5]. This strategy aims to improve healthcare practices and outcomes by integrating multiple and synergistic interventions [6].

Hand hygiene practices can be evaluated using a WHO-standardized observation tool [7], which outlines five opportunities or ‘moments’ for observing hand hygiene within healthcare settings. These opportunities include performing hand hygiene before and after contact with patients [7].

The WHO observation tool has been used in various countries and has shown different levels of hand hygiene compliance in the Eastern Mediterranean area. Compliance was 52.8% in Egypt, 32.3% in Tunisia, 18.6% in Algeria, and 16.9% in Morocco [8]. Several before-and-after studies from different countries, including those from Tunisia, have shown improvements in hand hygiene knowledge and practices after the introduction of the WHO multimodal strategy [9,10]. However, these assessments were typically conducted within a period of two years of introducing the strategy.

A PubMed review revealed no studies evaluating the longer-term impact of the WHO multimodal strategy on hand hygiene, specifically, changes over a period of up to five years. Furthermore, there are no studies from overcrowded and understaffed South Tunisian University hospitals, where hand hygiene standards are expected to be higher due to the elevated healthcare demands and challenges in these settings.

This study’s objectives were to compare knowledge and compliance of healthcare practitioners (HCPs) to hand hygiene practice in relation to the five moments for hand hygiene before (2019) and after (2023) the implementation of the WHO multimodal strategy in two University Hospitals in the Sfax Governate of Tunisia.

## 2. Materials and Methods

### 2.1. Study Design

A before-and-after cross-sectional assessment using a WHO observational tool for hand hygiene.

### 2.2. Study Setting

- General setting

Tunisia is located in Africa and is part of the 20 Arab countries, with an estimated population of 11.7 million inhabitants [11]. The governorate of Sfax lies in the coastal region of South Eastern Tunisia and has a population of 1,022,900, accounting for 9% and 48.7% of the total population in Tunisia and Southern Tunisia, respectively [12]. There are a total of 110 district hospitals (first level), 31 regional hospitals (second level), and 32 university hospitals (third level) in the country.

- Specific Setting

The study period was from January 2019 to December 2023. The study sites were the two University Hospitals (third level), Hedi Chaker and Habib Bourguiba, in the governorate of Sfax, Southern Tunisia.

The Hedi Chaker University Hospital has 182 physicians and 579 paramedical staff. It has 957 beds distributed in 24 departments (20 medical departments, 2 surgical departments, and 2 intensive care and emergency departments).

The Habib Bourguiba University Hospital has 250 physicians and 1013 paramedical staff. The hospital has 21 departments (3 intensive care and emergency departments, 9 medical departments, and 9 surgical departments).

### 2.3. Implementation of the WHO Multimodal Strategy

**Figure 1:**
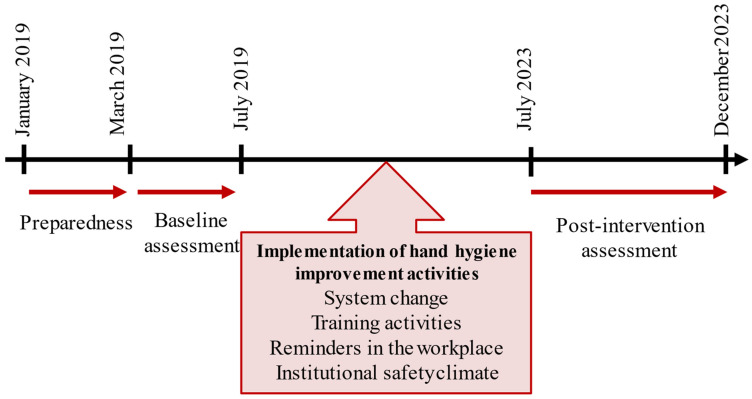
The steps of implementation of the WHO multimodal strategy on hand hygiene in the two university hospitals of Southern Tunisia (2019–2023).

Figure 1 shows the four steps in implementing the WHO multimodal strategy over a period of five years (2019–2023). Included in the implementation of the WHO multimodal strategy is a baseline evaluation for establishing the knowledge of the current situation. Subsequent follow-up evaluations, then periodically assess the impact of activities of hand hygiene.

- **Step 1. Preparedness**

From January to March 2019, the Department of Hospital Hygiene ensured preparedness by reviewing the main challenges and providing the necessary infrastructure and resources. Infection Prevention Control (IPC) committees were formally established in the two university hospitals in 2020. Each department had an IPC referent person appointed by the IPC committee to support the implementation of the IPC program according to the national guidelines [5].

- **Step 2. Baseline evaluation**

A trained IPC implementation team, including an IPC specialist and hospital hygiene doctors, conducted a baseline assessment of hand hygiene knowledge and practice over three months (April to June 2019). The WHO observation tool was used [13]. In order to prevent the Hawthorne effect, the observations were carried out without HCPs being aware [14].

- **Step 3. Implementation of hand hygiene improvement activities**

These specific activities started in July 2019 and continued yearly through 2023, as shown in Figure 1 [5]. The activities included the following:

**System change:** In order to ensure that the necessary infrastructure was in place, we used a monthly checklist to verify the availability and functionality at the point-of-care of alcohol-based hand rub, hand wash stations, running taps, liquid soap, hand towels, and gloves.

**Training activities:** A training program was established for all HCPs and tailored to bridge knowledge gaps identified in the baseline assessment.

**Reminders in the workplace:** Hand hygiene leaflets and posters were prepared and distributed to each department to be displayed at points-of-care and prominent areas in hospital departments.

**Institutional safety climate**: We encouraged an environment that motivates HCPs and promotes patient safety.

- **Step 4. Follow-up evaluation**

This included the period from August to December 2023. In December 2023, a second assessment was carried out by the investigators using the same hand hygiene observational tools and involving the same HCPs who participated in the baseline evaluation.

### 2.4. Definition of Terms

The five indications or moments for hand hygiene are based on those defined by the WHO guidelines [5]. A moment or indication for hand hygiene is when there is a perceived or actual risk of pathogen transmission from one surface to another via the HCP’s hands (gloved or ungloved) during successive tasks. According to WHO guidelines, the hands should be washed with soap or rubbed with alcoholic disinfectant in five moments:

- Moment 1: before patient contact;
- Moment 2: before a procedure or an aseptic task;
- Moment 3: after a body fluid exposure risk;
- Moment 4: after touching a patient;
- Moment 5: after touching the patient’s surroundings.

An opportunity for hand hygiene action is a situation when one of the moments for hand hygiene is present and observed during patient care. Hand hygiene compliance was calculated as the proportion of hand hygiene indications for which HCPs performed a correct action.

### 2.5. Study Population and Sample Size

At the onset of implementing the multimodal strategy, we compiled an exhaustive list of medical and paramedical staff from both hospitals to identify individuals who would participate in the initiative. Using a randomized sampling approach, stratified by hospital departments (medical, surgical, and intensive care), individuals were selected to ensure a balanced representation. Sampled individuals were informed of the multimodal strategic plan and consented to participate in both the baseline and follow-up assessments of hand hygiene knowledge and practice. Consequently, the same medical and paramedical HCPs who were involved in the baseline evaluation participated in the subsequent assessments.

The sample size for HCPs’ knowledge was 196, using an 80% statistical power, 0.05 alpha risk of error, a 5% margin of error, and an expected percentage of change in the hand hygiene knowledge equal to 15% [15,16]. To account for possible non-responses and HCPs who may have changed jobs and moved to another hospital, we included an additional 20%, making up a total of 235 HCPs.

The sample size for hand hygiene practices was set at 1376 observations, calculated based on WHO recommendations [17], a prior study conducted in Tunisia [18], and with an estimated improvement of hand hygiene practices from 32.1% to 39% between the before and after assessment. To account for possible non-responses and HCPs who may have changed jobs and moved to another hospital, an additional 20% was added, resulting in a total of 1652 observations.

### 2.6. Data Variables and Sources of Data

Data variables included 25 questions from the WHO Hand Hygiene Knowledge Questionnaire for Healthcare Workers that assessed knowledge on hand hygiene in 2019 and 2023. These questions were focused on three key areas: (a) the role of hands in transmission of HAIs, (b) hand hygiene moments or indications; and (c) proper hand hygiene techniques. Additional information was collected on gender, HCP categories, specialty of the affiliated departments, and routine use of alcohol-based hand rub [13]. For assessing hand hygiene practices, we used the WHO Observation form, which included the five opportunities for hand hygiene, hand hygiene actions performed, and hand hygiene techniques [7].

### 2.7. Analysis and Statistics

Data was entered, coded, and analyzed using SPSS Statistics for Windows, Version 20.0 (IBM SPSS Statistics for Windows, Version 20.0. Armonk, NY, USA: IBM Corp.). Categorical variables were expressed as numbers and percentages.

To assess hand hygiene knowledge, we calculated the number and proportion of HCPs who responded correctly to each of the 25 questions and considered the responses to be satisfactory if at least 80% of the 25 questions were answered correctly. To compare knowledge before and after the implementation of the strategy, we used absolute change in overall knowledge of the cohort and expressed this as a percentage change with 95% confidence intervals.

Hand hygiene compliance was calculated by dividing the number of hand hygiene actions (numerator) by the total number of hand hygiene opportunities (denominator) and expressed as a percentage [19]. Differences between groups were compared using the chi-square test, with a *p*-value ≤ 0.05 considered statistically significant.

## 3. Results

### 3.1. Characteristics of the Study Population

A total of 246 HCPs participated in the before and after assessments. The majority were women (77%), worked in the medical department (67%), and were from the paramedical staff category (65%) (Table 1).

### 3.2. Knowledge of Healthcare Practitioners About Hand Hygiene

In 2019, a total of 42 (17%) HCPs reached the satisfactory threshold of ≥80% of questions answered correctly. By 2023, this number increased to 47 HCPs (19%).

Table 2 and Table 3 present a comparison of hand hygiene knowledge among HCPs, before and after implementing the WHO multimodal strategy. Knowledge on hand hygiene significantly improved for 10 out of 25 questions (in the WHO tool), the increase ranging from 12% to 38%. However, knowledge significantly declined for eight questions, with decreases ranging from 5% to 40%. Seven questions showed no significant changes in knowledge.

The areas where knowledge declined included (a) whether hand hygiene is required after exposure to body fluids; (b) whether hand washing and hand rubbing are recommended to be performed sequentially; (c) the appropriate hand hygiene technique to be used after emptying a bedpan, after removing examination gloves, after making a patient’s bed and after visible exposure to blood; (d) and whether wearing jewelry, artificial fingernails, and use of hand cream are associated with colonization of the hand with harmful germs.

### 3.3. Compliance of Healthcare Practitioners to Hand Hygiene Practice

Overall compliance with hand hygiene practice climbed from 21% at baseline (2019) to 40% in the follow-up assessment (2023) (*p* < 0.001). Significant improvements were observed across the different hospital departments (*p* < 0.001) and staff categories (*p* < 0.001). The lowest compliance was noted for two specific indications for hand hygiene: (a) before touching a patient, where compliance increased marginally from 23% in 2019 to 29% in 2023, and (b) before clean/aseptic procedures where compliance decreased from 52% in 2019 to 37% in 2023 (Table 4).

## 4. Discussion

This study demonstrates that the implementation of the WHO multimodal strategy resulted in a two-fold improvement in hand hygiene compliance, increasing from 21% in 2019 to 40% in 2023. Despite this encouraging progress, compliance fell short of the WHO’s ambitious 80% target. The findings also revealed eight specific areas where knowledge regressed.

This study underscores the challenges of achieving behavioral change, emphasizing the need for complementary measures to drive faster progress in hand hygiene compliance. From a public health standpoint, the overall observed improvement of 19% over a five-year timeline in our setting translated to a modest annual average improvement of about 4%, highlighting the slow pace of advancement in this critical area.

The study strengths include the same trained IPC team was used to assess knowledge and compliance during both comparison periods, thereby ensuring consistency in evaluation; the same cohort of healthcare providers was assessed, which enhanced the reliability of the findings; and the study adhered to STROBE guidelines for the reporting of observational studies in epidemiology [20]. Importantly, the study addressed an identified national operational research priority and is thus likely to influence policy and practice.

One of the main study limitations is that the assessment was conducted in only two university hospitals, which may limit the generalizability of the findings to other healthcare settings in the country, particularly rural or non-academic facilities. Furthermore, we did not explore the specific reasons for low levels of hand hygiene compliance among HCPs nor their perspectives on the challenges they probably faced in their work environments. Notably, it remains unclear whether compliance was influenced by workload and/or infrastructure or resource limitations, such as shortages of human resources, alcohol-based solutions, or hand-washing facilities. Understanding these factors would be crucial for driving substantial improvements in hand hygiene compliance. Thus, there is an urgent need for further qualitative research in these aspects.

This study’s findings have a number of policy and practice implications.

First, we identified several areas where HCPs knowledge of hand hygiene regressed. This decline poses a real risk, as gaps in knowledge may lead to non-compliance due to inadequate awareness. To counter this tendency, we recommend implementing targeted refresher training sessions for all HCPs that prioritize areas where knowledge has regressed. Additionally, we need to evaluate potential shortcomings in the training approach that may have contributed to this undesirable finding.

Second, the lowest levels of hand hygiene compliance were observed in situations aimed at protecting patients, such as before touching a patient and prior to performing clean/aseptic procedures. This may indicate a greater focus on “self-protection” by HCPs. A similar observation has been reported from other African hospitals [21,22,23,24]. Ultimately, this highlights a lack of awareness of the interlinkage between safe hands, safe healthcare environments, and the safety of healthcare providers themselves. The key message to be reinforced is that healthcare providers will only be truly safe when their interactions with patients and their environment are also safe.

Third, while the overall improvement in compliance is encouraging, it still falls short of the WHO’s ambitious target of 80% [5]. Although the COVID-19 pandemic may have positively impacted hand hygiene practices between 2020 and 2022, there is a possibility of subsequent apathy; however, our study does not allow us to confirm this hypothesis [25,26]. The 40% compliance seen in our study is marginally higher than similar reports from tertiary care facilities in Ethiopia, Kenya, and Nigeria, where compliance levels ranged from 17 to 30% [22,27,28,29,30]. A study from a tertiary hospital in Tunisia showed an increase in hand hygiene compliance from 32.1 to 39.4%, which is relatively similar to our study [18].

To accelerate progress in compliance, simply performing “more of the same” is unlikely to yield significant results; rather, we must focus on “doing things differently.” For example, we should explore innovative ways to motivate healthcare providers and actively engage them in addressing their challenges. Regular evaluations, along with ensuring adequate staff availability and monitoring workload, are critical, as HCPs may be overwhelmed by competing priorities, which may compromise hand hygiene. In addition, during the five years of this study, there was a moratorium on staff employment due to economic restrictions [31]. This might imply that while HCPs workload increased, human resource numbers remained static.

Finally, to drive effective behavioral change, several adjunctive measures could be considered such as: (a) Strengthening IPC with financial and dedicated human resources, as current IPC efforts are largely voluntary and often an ‘add on’ responsibility on the shoulders of busy HCPs; (b) implementing a balanced reallocation of HCPs between departments or services experiencing high workloads, which may foster a more conducive attitude towards hand hygiene compliance; and (c) introducing recognition and reward incentives for improvements in hand hygiene practices, such as certifications, quality service stars and performance-based financial incentives. Interdepartmental competitions for positive reinforcement and addressing a combination of determinants (social influence, attitude, self-efficiency or intention) may also be considered [32,33]. Other interventions could include the installation of voice reminder devices, which had a positive impact on HCPs’ attitudes and compliance in Nigeria [34]. Such approaches may prove more effective than static reminders pasted on the walls of health facilities, as attention may wane over time. Finally, sustained hand hygiene activities, as has been shown in Finland over seven years, can produce a significant impact [35].

## 5. Conclusions

The implementation of the WHO multimodal strategy in two University hospitals in Southern Tunisia led to a significant improvement in hand hygiene compliance, with a notable two-fold increase. Despite this progress, the compliance still fell well short of the WHO’s 80% target, highlighting the need for adjunctive interventions to accelerate progress in hand hygiene outcomes. The findings highlight areas for improvement and can inform targeted interventions. However, it is important to emphasize that this is a local-regional study, and the results primarily reflect the context and practices of the two hospitals analyzed. While the data are highly relevant for guiding institutional policies and quality improvement efforts within these settings, generalizing the findings to other hospitals or regions should be approached with caution.

## Figures and Tables

**Table 1:** Characteristics of healthcare professionals who were assessed on knowledge about hand hygiene before and after the implementation of the WHO multimodal strategy, South Tunisian University Hospitals, (2019–2023).

Characteristics	Number	(%)
**Total**	246	(100)
**Gender**		
Male	56	(23)
Female	190	(77)
**Departments**		
Medical	164	(67)
Surgical	43	(17)
Intensive care	39	(16)
**Staff category**		
Medical	86	(35)
Paramedical	160	(65)

**Table 2:** Comparison of correct responses to knowledge (on the role of hands in the transmission of germs and hand hygiene indications) among healthcare professionals (N = 246), before (2019) and after (2023) implementing the WHO multimodal strategy, South Tunisian University Hospitals.

	Correct Answers in 2019		Correct Answers in 2023		% Change (95% CI *)
Questions	n	(%)	n	(%)	
**Which of the following is the main route of cross-transmission in a healthcare facility?**					
Healthcare workers’ hands when not clean (correct)	166	(67)	207	(84)	17 (10 to 24)
**What is the most frequent source of germs responsible for healthcare-associated infections?**					
Germs are already present on or within the patient (correct)	83	(34)	153	(62)	28 (20 to 36)
**Which of the following hand hygiene actions prevents transmission of germs to the patient?**					
Before touching a patient (correct)	191	(78)	228	(93)	15 (9 to 21)
Immediately after a risk of body fluid exposure (incorrect)	109	(44)	95	(39)	−5 (−14 to 4)
Immediately before a clean/aseptic procedure (correct)	114	(46)	97	(40)	−6 (−15 to 3)
After exposure to the immediate surroundings of a patient (incorrect)	160	(65)	217	(88)	23 (16 to 30)
**Which of the following hand hygiene actions prevents transmission of germs to the healthcare worker?**					
After touching a patient (correct)	194	(79)	223	(91)	12 (6 to 18)
Immediately after a risk of body fluid exposure (correct)	156	(63)	217	(88)	25 (18 to 32)
Immediately before a clean/aseptic procedure (incorrect)	163	(66)	217	(88)	22 (15 to 29)
After exposure to the immediate surroundings of a patient (correct)	144	(58)	149	(61)	3 (−6 to 12)

* CI: confidence interval.

**Table 3:** Comparison of correct responses to questions on knowledge about hand hygiene techniques among healthcare professionals (N = 246), before (2019) and after (2023) implementing the WHO multimodal strategy, South Tunisian University Hospitals.

	Correct Answers in 2019		Correct Answers in 2023		% Change (95% CI *)
Questions	(n)	(%)	(n)	(%)	
**Which of the following statements on alcohol-based hand rub and hand washing with soap and water are true?**					
Hand rubbing is more rapid for hand cleansing than hand washing (correct)	158	(64)	192	(78)	14 (6 to 22)
Hand rubbing causes skin dryness more than hand washing (incorrect)	78	(32)	123	(50)	18 (9 to 27)
Hand rubbing is more effective against germs than hand washing (correct)	71	(29)	165	(67)	38 (30 to 46)
Hand washing and hand rubbing are recommended in sequence (incorrect)	134	(54)	73	(30)	−24 (−32 to −16)
Minimal time needed for alcohol-based hand rub to kill most germs on your hands 20 s (correct)	141	(57)	179	(73)	16 (8 to 24)
**Which type of hand hygiene method is required in the following situations?**					
Before palpation of the abdomen (Rubbing)	220	(90)	231	(94)	4 (−1 to 9)
Before giving an injection (Rubbing)	241	(98)	237	(96)	−2 (−5 to 10)
After emptying a bedpan (Rubbing)	216	(88)	118	(48)	−40 (−47 to −33)
After removing examination gloves (Rubbing)	196	(80)	119	(48)	−32 (−40 to −24)
After making a patient’s bed (Rubbing)	244	(99)	232	(94)	−5 (−8 to −2)
After visible exposure to blood (Washing)	215	(87)	182	(74)	−13 (−20 to −6)
**Which of the following should be avoided because of being associated with a likelihood of colonization of hands with harmful germs?**					
Wearing jewelry (correct)	230	(93)	209	(85)	−8 (−13 to −3)
Damaged skin (correct)	178	(72)	152	(62)	−10 (−18 to −2)
Artificial fingernails (correct)	222	(90)	219	(89)	−1 (−6 to 4)
Regular use of a hand cream (incorrect)	164	(67)	101	(41)	−26 (−35 to −17)

* CI: confidence interval.

**Table 4:** Comparison of hand hygiene compliance among healthcare professionals concerning the opportunity (moment) for hand hygiene action, before (2019) and after (2023) implementing the WHO multimodal strategy, Tunisian University Hospitals.

	Before (2019)			After (2023)			% Change	*p*-Value
	Opportunities for HH Action (n)	HH Action Performed (n)	Compliance * (%)	Opportunities for HH Action (n)	HH Action Performed (n)	Compliance * (%)		
**Overall**	832	171	21	865	344	(40)	19 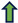	<0.001
**Indications for hand hygiene**								
Before touching a patient	227	53	(23)	254	75	(29)	6 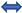	0.1
Before clean/aseptic procedure	29	15	(52)	35	13	(37)	−15 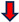	0.2
After body fluid exposure risk	57	17	(30)	80	48	(60)	30 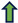	<0.001
After touching a patient	141	52	(37)	158	75	(47)	10 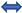	0.06
After touching the patient’s surroundings	378	34	(9)	338	133	(39)	30 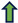	<0.001
**Department**								
Medical	549	123	(22)	582	218	(37)	15 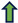	<0.001
Surgical	144	34	(24)	153	53	(34)	10 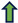	0.004
Intensive care	139	14	(10)	130	73	(56)	46 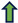	<0.001
**Staff category**								
Medical	271	45	(17)	320	126	(39)	22 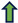	<0.001
Paramedical	561	126	(22)	545	218	(40)	18 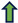	<0.001

* Compliance rate (%): the number of hand hygiene actions performed divided by the number of opportunities and multiplied by 100. HH = hand hygiene; HH action = alcohol-based hand rub or hand wash; percentages are row percentages. The *p* values represent comparisons of hand hygiene actions between each period carried out using chi-square tests.

## Data Availability

Requests to access these data should be sent to the corresponding author.

## Abbreviations

The following abbreviations are used in this manuscript:

HAIs	Healthcare-associated infections
WHO	World Health Organization
HCPs	Healthcare practitioners
IPC	Infection Prevention Control
CI	Confidence interval

## References

[1] Harun M.G.D., Anwar M.M.U., Sumon S.A., Mohona T.M., Hassan M.Z., Rahman A., Abdullah S.A.H.M., Islam M.S., Oakley L.P., Malpiedi P. (2023). Hand hygiene compliance and associated factors among healthcare workers in selected tertiary-care hospitals in Bangladesh. J. Hosp. Infect..

[2] WHO|Member States Information Session and GB Briefings. https://apps.who.int/gb/mspi/.

[3] Improving Hand Hygiene Compliance in the Emergency Department: Getting to the Point|BMC Infectious Diseases. https://bmcinfectdis.biomedcentral.com/articles/10.1186/1471-2334-13-367.

[4] Pittet D., Hugonnet S., Harbarth S., Mourouga P., Sauvan V., Touveneau S., Perneger T.V. (2000). Effectiveness of a hospital-wide programme to improve compliance with hand hygiene. Infection Control Programme. Lancet.

[5] A Guide to the Implementation of the WHO Multimodal Hand Hygiene Improvement Strategy. https://www.who.int/publications/i/item/a-guide-to-the-implementation-of-the-who-multimodal-hand-hygiene-improvement-strategy.

[6] Sandbekken I.H., Utne I., Hermansen Å., Grov E.K., Løyland B. (2024). Impact of multimodal interventions targeting behavior change on hand hygiene adherence in nursing homes: An 18-month quasi-experimental study. Am. J. Infect. Control.

[7] Monitoring Tools WHO: Additional Hand Hygiene Evaluation Tools. https://www.who.int/teams/integrated-health-services/infection-prevention-control/hand-hygiene/monitoring-tools.

[8] Amazian K., Abdelmoumene T., Sekkat S., Terzaki S., Njah M., Dhidah L., Caillat-Vallet E., Saadatian-Elahi M., Fabry J. (2006). Multicentre study on hand hygiene facilities and practice in the Mediterranean area: Results from the NosoMed Network. J. Hosp. Infect..

[9] Bajunaid R.M., Saeed A., Bostaji M., Farsi N.J. (2024). Hand hygiene compliance and improvement interventions in the Eastern Mediterranean Region: A systematic review and meta-analysis. Infect. Prev. Pract..

[10] Mlouki I., Ayed S.B., Chebbi F., Rezg N., Khouildi A., Sassi A.H., El Mhamdi S. (2023). Hand hygiene and biomedical waste management among medical students: A quasi-experimental study evaluating two training methods. BMC Med. Educ..

[11] The Global Compact on Refugees Human Development Report 2021/2022. http://globalcompactrefugees.org/human-development-report-20212022.

[12] Tunisia Carte-Sanitaire-Synthse. http://www.sicad.gov.tn/Fr/Services-en-ligne_64_7_D5622.

[13] (2009). WHO Hand Hygiene Knowledge Questionnaire for Health-Care Workers. https://www.google.com/search?q=Hand+hygiene+knowledge+questionnaire+for+health-care+workers.+WHO%3B+2009.&oq=Hand+hygiene+knowledge+questionnaire+for+health-care+workers.+WHO%3B+2009.&gs_lcrp=EgZjaHJvbWUyBggAEEUYOdIBCTE4NDZqMGoxNagCCLACAQ&sourceid=chrome&ie=UTF-8.

[14] Chen L.F., Weg M.W.V., Hofmann D.A., Reisinger H.S. (2015). The Hawthorne Effect in Infection Prevention and Epidemiology. Infect. Control Hosp. Epidemiol..

[15] Rollins School of Public Health. https://sph.emory.edu/index.html.

[16] Karaoglu M.K., Akin S. (2018). Effectiveness of Hygienic Hand Washing Training on Hand Washing Practices and Knowledge: A Nonrandomized Quasi-Experimental Design. J. Contin. Educ. Nurs..

[17] Sax H., Allegranzi B., Chraïti M.N., Boyce J., Larson E., Pittet D. (2009). The World Health Organization hand hygiene observation method. Am. J. Infect. Control.

[18] Ben Fredj S., Ben Cheikh A., Bhiri S., Ghali H., Khefacha S., Dhidah L., Merzougui L., Ben Rejeb M., Said Latiri H. (2020). Multimodal intervention program to improve hand hygiene compliance: Effectiveness and challenges. J. Egypt. Public. Health Assoc..

[19] World Health Organization, WHO Patient Safety (2009). WHO Guidelines on Hand Hygiene in Health Care. https://www.who.int/publications/i/item/9789241597906.

[20] von Elm E., Altman D.G., Egger M., Pocock S.J., Gøtzsche P.C., Vandenbroucke J.P. (2007). STROBEInitiative Strengthening the Reporting of Observational Studies in Epidemiology (STROBE) statement: Guidelines for reporting observational studies. BMJ.

[21] Antimicrobial Resistance Collaborators (2022). Global burden of bacterial antimicrobial resistance in 2019: A systematic analysis. Lancet.

[22] Federal Medical Centre Gu O. (2015). Five Moments for Hand Hygiene: A Study of Compliance among Healthcare Workers in a Tertiary Hospital in South East Nigeria. Community Med. Public. Health Care.

[23] Müller S.A., Diallo A.O.K., Wood R., Bayo M., Eckmanns T., Tounkara O., Arvand M., Diallo M., Borchert M. (2020). Implementation of the WHO hand hygiene strategy in Faranah regional hospital, Guinea. Antimicrob. Resist. Infect. Control.

[24] Abuosi A.A., Akoriyea S.K., Ntow-Kummi G., Akanuwe J., Abor P.A., Daniels A.A., Alhassan R.K. (2020). Hand hygiene compliance among healthcare workers in Ghana’s health care institutions: An observational study. J. Patient Saf. Risk Manag..

[25] Kanaujia R., Biswal M., Kaur K., Kaur H., Kaur R., Kaur H., Kaur M., Arora P., Dhaliwal N. (2024). Hand hygiene compliance of respiratory physiotherapists: An analysis of trends over eight years including the COVID-19 pandemic period. Indian. J. Med. Microbiol..

[26] Higashionna T., Hagiya H., Fujita Y., Kiguchi T. (2024). Trends in the hand hygiene practices using alcohol-based hand rubs in Japanese hospitals before and after the novel coronavirus pandemic: An observational study using national surveillance data. J. Hosp. Infect..

[27] Omuemu V., Ogboghodo E.O., Opene R.A., Oriarewo P. (2013). Hand hygiene practices among doctors in a tertiary health facility in southern Nigeria. J. Med. Trop..

[28] Onyedibe K.I., Shehu N.Y., Pires D., Isa S.E., Okolo M.O., Gomerep S.S., Ibrahim C., Igbanugo S.J., Odesanya R.U., Olayinka A. (2020). Assessment of hand hygiene facilities and staff compliance in a large tertiary health care facility in northern Nigeria: A cross sectional study. Antimicrob. Resist. Infect. Control.

[29] Tesfaye G., Gebrehiwot M., Girma H., Malede A., Bayu K., Adane M. (2022). Application of the gold standard direct observation tool to estimate hand hygiene compliance among healthcare providers in Dessie referral hospital, Northeast Ethiopia. Int. J. Environ. Health Res..

[30] Kiprotich K., Wang H., Kaminga A.C., Kessi M. (2021). Observed and self-reported hand hygiene compliance and associated factors among healthcare workers at a county referral hospital in Kenya. Sci. Afr..

[31] (2024). Health workforce in the Eastern Mediterranean Region: From COVID-19 lessons to actions|Request, P.D.F. https://www.researchgate.net/publication/377072055_Health_workforce_in_the_Eastern_Mediterranean_Region_From_COVID-19_lessons_to_actions.

[32] Lotfinejad N., Assadi R., Aelami M.H., Pittet D. (2020). Emojis in public health and how they might be used for hand hygiene and infection prevention and control. Antimicrob. Resist. Infect. Control.

[33] Huis A., van Achterberg T., de Bruin M., Grol R., Schoonhoven L., Hulscher M. (2012). A systematic review of hand hygiene improvement strategies: A behavioural approach. Implement. Sci..

[34] Ibrahim U.M., Gajida A.U., Garba R.M., Gadanya M.A., Umar A.A., Jalo R.I., Adamu A.L., Ismai F., Tsiga-Ahmed, Gwarzo D.H. (2021). Effect of voice reminder on compliance with recommended hand hygiene practise among health-care workers in Kano metropolis. Niger. Postgrad. Med. J..

[35] Ojanperä H., Ohtonen P., Kanste O., Syrjälä H. (2022). Impact of direct hand hygiene observations and feedback on hand hygiene compliance among nurses and doctors in medical and surgical wards: An eight-year observational study. J. Hosp. Infect..

